# Flux Enhancement in Hybrid Pervaporation Membranes Filled with Mixed Magnetic Chromites ZnCr_2_Se_4_, CdCr_2_Se_4_ and CuCr_2_Se_4_

**DOI:** 10.3390/molecules30244784

**Published:** 2025-12-15

**Authors:** Łukasz Jakubski, Izabela Jendrzejewska, Artur Chrobak, Klaudiusz Gołombek, Gabriela Dudek

**Affiliations:** 1 Department of Physical Chemistry and Technology of Polymers, PhD School, Silesian University of Technology, Strzody 9, 44-100 Gliwice, Poland; 2 Department of Physical Chemistry and Technology of Polymers, Faculty of Chemistry, Silesian University of Technology, Strzody 9, 44-100 Gliwice, Poland; 3 Institute of Chemistry, University of Silesia in Katowice, 40-007 Katowice, Poland; 4 Institute of Physics, University of Silesia in Katowice, 75 Pułku Piechoty 1A, 41-500 Chorzow, Poland; 5 Materials Research Laboratory, Faculty of Mechanical Engineering, Silesian University of Technology, Konarskiego 18A, 44-100 Gliwice, Poland

**Keywords:** pervaporation, ethanol dehydration, hybrid magnetic membranes, zinc, cadmium, copper chromite selenides, low-emission fuel processing

## Abstract

The integration of bioethanol into transportation fuels requires efficient purification methods to overcome the ethanol–water azeotrope, which cannot be separated by conventional distillation. Pervaporation has become an attractive alternative, offering high selectivity while minimising energy consumption. To further improve membrane performance, this study analyses sodium alginate-based hybrid membranes containing binary mixtures of chromite selenides with varying magnetic properties (ZnCr_2_Se_4_, CdCr_2_Se_4_, and CuCr_2_Se_4_). Pairwise combinations of these fillers were introduced to create complex magnetic structures that can influence polymer–filler interactions and molecular transport. Structural, magnetic, and functional characterisation showed that membrane properties were strongly dependent on the type and proportion of fillers. In particular, the CdCr_2_Se_4_ with CuCr_2_Se_4_ combination exhibited the most favourable balance between permeation flux and selectivity, achieving the highest parameters, including pervaporation separation index (PSI) reaching 747 kg·m^−2^·h^−1^. This superior performance is attributed to the synergistic interaction of these two magnetic fillers, which enhances membrane selectivity while maintaining its integrity. This work presents a novel approach to membrane-based separation, advancing the development of energy-efficient, environmentally sustainable bioethanol purification technologies.

## 1. Introduction

The integration of bioethanol into conventional fuel formulations has emerged as a strategic and environmentally sustainable approach to mitigating harmful emissions and enhancing combustion efficiency in the transportation sector [1,2,3]. When used as a fuel additive, anhydrous ethanol significantly reduces the emission of pollutants, including carbon oxides (COx), volatile organic compounds (VOCs), nitrogen oxides (NOx), and fine particulate matter (PM2.5) [4,5]. Such improvements not only alleviate the adverse environmental impacts of fossil fuel combustion but also make a significant contribution to global initiatives to improve air quality and reduce atmospheric pollution [6]. Moreover, the incorporation of ethanol into fuel blends aligns with the ongoing transition toward renewable and low-emission energy sources, thereby reinforcing its role in the development of sustainable energy systems [7,8,9]. Consequently, the demand for high-purity ethanol, particularly in its anhydrous form, continues to rise in response to increasingly stringent environmental regulations and air quality standards [10,11]. One of the primary technical challenges in ethanol purification is separating the ethanol–water azeotropic mixture, which cannot be efficiently achieved with conventional distillation methods. In this context, pervaporation has gained recognition as an innovative and energy-efficient membrane-based separation technology [12,13]. Its principal advantages—superior selectivity, reduced energy consumption, and compatibility with continuous industrial operations-make it an up-and-coming technique for fuel-grade ethanol enrichment. These attributes position pervaporation as a compelling solution in the evolving field of sustainable, environmentally conscious chemical engineering [14].

To improve the efficiency and selectivity of pervaporation membranes, research is increasingly focusing on the development of hybrid materials that incorporate inorganic fillers into polymer matrices [15,16,17]. This approach leverages the advantageous properties of biopolymers, such as excellent film-forming ability, biodegradability, and inherent hydrophilicity, while incorporating the functional benefits of inorganic additives. The inclusion of such fillers can modify the polymer network by creating additional transport pathways, altering the membrane morphology, or adjusting its free volume and chain mobility. As a result, these structural modifications collectively improve transport properties and enhance the separation performance of the membrane system.

In this study, a novel approach is proposed involving the pairwise incorporation of magnetically distinct selenide chromites: ZnCr_2_Se_4_, which exhibits a spiral antiferromagnetic structure with a Néel temperature of approximately 21 K; CdCr_2_Se_4_, characterised by its ferromagnetic nature and a Curie temperature below 130 K; and CuCr_2_Se_4_, a metallic ferromagnet with a Curie temperature of about 430 K [18]. On the one hand, this group of compounds exhibits a correlated electron system; on the other hand, magnetic frustration increases the system’s complexity, inducing phenomena such as spin glass, colossal magnetoresistance, and the magnetoelectric effect [19]. The chromium-based spinels ACr_2_Cr_4_ (A = Cd, Zn, Cu) crystallise in the cubic symmetry with the space group Fd3m, which can be described as a cubic close-packed arrangement of anions, within which the A-cations occupy one-eighth of the tetrahedral voids and Cr-ions in half of the octahedral voids. Consequently, each unit cell comprises eight formula units amounting to 56 ions in total, including 8 A^2+^, 16 Cr^3+^, and 32 Se^2−^ ions [20]. The magnetic behaviour of these compounds depends on exchange magnetic interactions between Cr-Cr competing with each other. Interaction Cr-X-Cr causes ferromagnetic ordering, whereas the Cr-X-X-Se and Cr-X-A-X-Cr interactions arrange antiferromagnetic alignment of Cr^3+^ magnetic moments [21]. Given the combination of their structural flexibility, relatively large unit-cell size (above 10 Å), and the presence of many intrinsic vacancies, together with their characteristic magnetic properties, we hypothesised that mixtures of these spinels could be used as promising fillers for alginate membranes. The incorporation of such selenide-chromite pairs into a single membrane matrix generates a complex internal magnetic architecture that can influence molecular diffusion, filler-polymer interactions, and, potentially, the alignment or orientation of functional particles within the material. To date, the combination of an antiferromagnetic and a ferromagnetic component, unlike in more common ferromagnetic–ferromagnetic systems, has not been demonstrated within a single pervaporation membrane, marking a significant innovation in membrane design. This dual-filler concept not only opens new avenues for tailoring the magnetic and physicochemical characteristics of hybrid membranes but also provides a foundation for exploring how contrasting magnetic behaviours may synergistically enhance selective mass transport processes.

In this work, we report on the fabrication and characterisation of sodium alginate-based hybrid membranes modified with binary mixtures of ZnCr_2_Se_4_ and CdCr_2_Se_4_, ZnCr_2_Se_4_ and CuCr_2_Se_4_, or CdCr_2_Se_4_ and CuCr_2_Se_4_. The primary objective of this study is to evaluate the structural, magnetic, and functional performance of these membranes for ethanol dehydration via pervaporation. Particular attention is devoted to examining how varying filler ratios influence key performance parameters, including permeate flux, separation factor, and pervaporation separation index (PSI).

The obtained results provide new insights into the role of magnetic interactions in enhancing membrane performance and highlight the potential of such hybrid systems for optimising ethanol purification. Overall, this study contributes to the advancement of environmentally sustainable technologies that promote cleaner fuel production and help mitigate pollutant emissions, aligning with the broader goals of sustainable development and improved air quality.

## 2. Results and Discussion

### 2.1. Morphological Analysis

**Figure 1:**
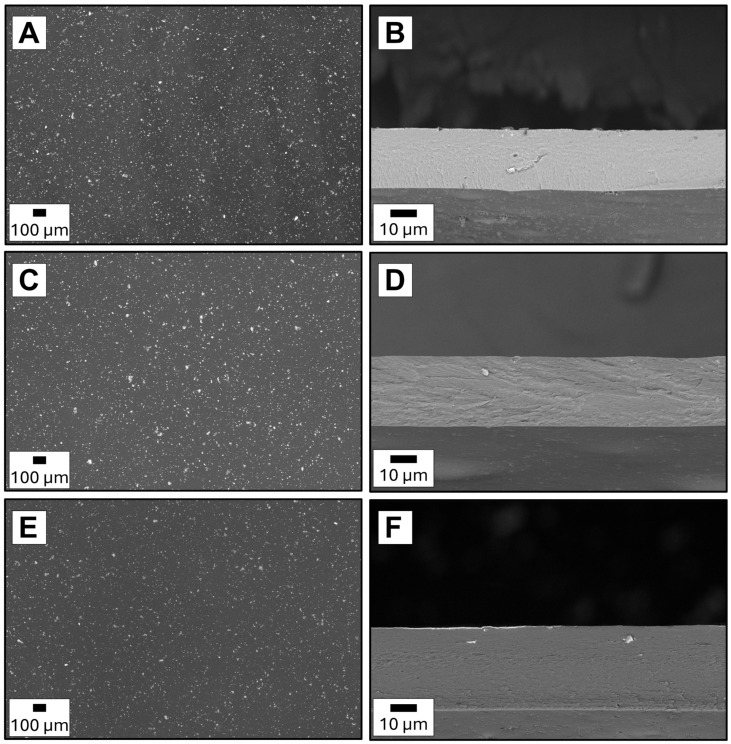
SEM images of cross-sections and surfaces of (**A**,**B**) 4Cu.1Cd, (**C**,**D**) 1Zn.4Cu and (**E**,**F**) 4Cd.1Zn membranes, respectively.

Figure 1 compares membrane morphologies obtained for different hybrid filler compositions, revealing distinct variations in the distribution of chromite particles within the alginate matrix. In the SEM images, the fillers appear as bright regions against the darker polymer background, enabling a precise evaluation of dispersion quality and particle distribution. The symbols of the membrane used in the manuscript are explained in Table 1. The 4Cu.1Cd (Figure 1A,B) exhibits a remarkably homogeneous morphology, with chromite particles uniformly dispersed and evenly embedded throughout the polymer matrix. The resulting structure appears continuous and defect-free, both in the cross-section and on the membrane surface.

In contrast, both membranes containing zinc chromite exhibit less homogeneous structures. In the 1Zn.4Cu and 4Cd.1Zn samples, clusters of aggregated particles are observed, resulting in localised filler-rich regions separated by filler-poor zones. In the case of the 4Cd.1Zn (Figure 1E,F), the filler-poor areas are larger and more pronounced, whereas in 1Zn.4Cu (Figure 1C,D), they are smaller but accompanied by more substantial particle agglomerates. This heterogeneity is evident not only in the cross-sections, where particle clusters disrupt the continuity of the polymer matrix, but also on the surface, which appears rougher and occasionally reveals exposed filler particles. Similar clustering phenomena have been reported in previous studies on mixed-metal oxides, where limited compatibility between the filler and polymer matrix hindered uniform dispersion [22].

These morphological characteristics are fundamental when correlating membrane structure with separation performance. For instance, Burts et al. [23] demonstrated that the incorporation of Al_2_O_3_·SiO_2_ nanoparticles into the selective layer of PVA/PAN membranes altered the surface morphology and increased both the thickness and hydrophilicity of the selective layer, as revealed by SEM and AFM analyses. These modifications reduced permeate flux but significantly enhanced the ethanol/water separation factor of the nanocomposite membranes. Similarly, in our previous work [24], the morphology of alginate membranes containing magnetite and molecular magnets was found to significantly influence mass transport during ethanol dehydration. The addition of magnetite promoted the formation of magnetic channels, facilitating water diffusion and increasing flux. The membrane exhibiting the best separation performance highlighted the critical role of filler distribution and channel formation in determining pervaporation separation factors.

In the present study, similar effects are expected. The continuous polymer phase observed in 4Cu.1Cd supports uniform water transport, helping to maintain the membrane’s stability. In contrast, the clustering and partial surface exposure observed in the zinc-containing membranes are likely to form dense domains or microdefects that act as less-selective channels for water transport. From a morphological perspective, the high degree of homogeneity of 4Cu.1Cd exhibits a clear structural advantage, in agreement with previous reports that emphasise the relevant role of filler dispersion in determining the effectiveness of hybrid membranes [18,24,25,26].

**Figure 2:**
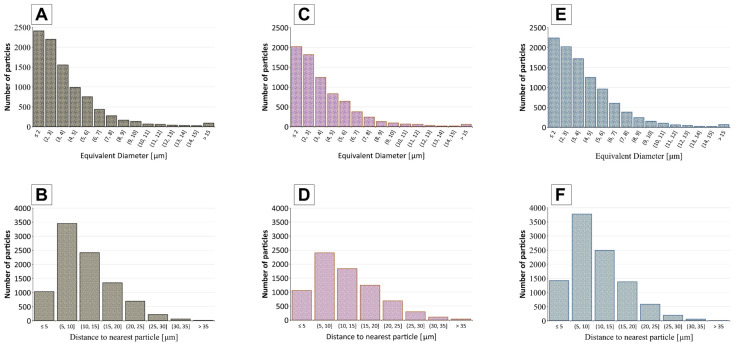
Distribution of particle sizes (**A**,**C**,**E**) and nearest neighbour (**B**,**D**,**F**) particle distances in membranes: (**A**,**B**) 4Cu.1Cd, (**C**,**D**) 1Zn.4Cu, (**E**,**F**) 4Cd.1Zn.

Figure 2 quantitatively confirms the above observations by analysing filler particle size distributions and particle distance for select membranes. The 4Cu.1Cd membrane (Figure 2A,B) shows a narrow particle size distribution, indicating that most embedded chromite particles are consistently small. Moreover, the nearest neighbour distance histogram for 4Cu.1Cd shows relatively large and uniformly distributed spacings between particles. In practical terms, this means that filler particles in the 4Cu.1Cd matrix remain mostly isolated from one another, each surrounded by a layer of polymer that prevents direct particle contact or the formation of large agglomerates. Such an arrangement ensures that the polymer phase is continuous and without large particle clusters. In contrast, the 1Zn.4Cu membrane (Figure 2C,D) displays a broader particle size distribution with a tail toward larger diameters, as well as a higher frequency of short particle distances. It suggests some degree of particle aggregation: many Cu and Zn filler particles are very close to each other or even touching, consistent with the SEM images showing cluster formation.

In 4Cd.1Zn (Figure 2E,F), the particle size distribution is between those of 4Cu.1Cd (A) and 1Zn.4Cu (C). The nearest-neighbour distance histogram resembles membrane 4Cu.1Cd (B) in its central tendency, but shows a higher fraction of very short distances, indicating local clustering. It agrees with the SEM images, which show areas devoid of particles adjacent to regions with densely packed particles. As a result, even though the average spacing is close to that of 4Cu.1Cd, the mix of short distances and empty areas seen in the SEM images shows that the particle distribution in 4Cd.1Zn is less uniform.

Comparable dispersion and aggregation patterns have been reported previously for magnetic nanoparticles embedded in polymer matrices. Shahdan et al. [27] observed that NiZn ferrite nanoparticles exhibited both well-dispersed and locally agglomerated regions within a PLA/LNR matrix, with the degree of aggregation varying according to processing parameters and nanoparticle concentration. Similarly, Eberbeck et al. [28] demonstrated that the aggregation behaviour of magnetic nanoparticles critically influences their functional performance across various technological applications. Through their modelling analysis, the authors found that nanoparticle clustering and scaling behaviour indicate when aggregates begin to form, and that these aggregates can significantly alter the material’s overall performance. Following these reports and our own morphological findings, we further analysed the magnetic filler particles to assess their aggregation tendencies and to evaluate their potential impact on the structural and functional properties of the obtained membranes.

Additionally, statistical parameters are provided in Table 1. The data show that all membranes exhibit some degree of particle agglomeration, as evidenced by small interparticle distances in each distribution. However, the extent of this effect clearly differs between the samples. The 4Cu.1Cd membrane has the narrowest particle size distribution and comparatively larger neighbour distances, indicating the most uniform dispersion among the three. The 1Zn.4Cu sample shows higher variability in both particle size and spacing, consistent with moderate aggregation. In contrast, the 4Cd.1Zn membrane displays the most remarkable heterogeneity, reflected by the highest standard deviation in neighbour distance (SD = 7.01 µm), which signifies the coexistence of densely packed regions and particle-depleted zones, in agreement with the SEM observations.

### 2.2. Magnetic Characteristics

**Figure 3:**
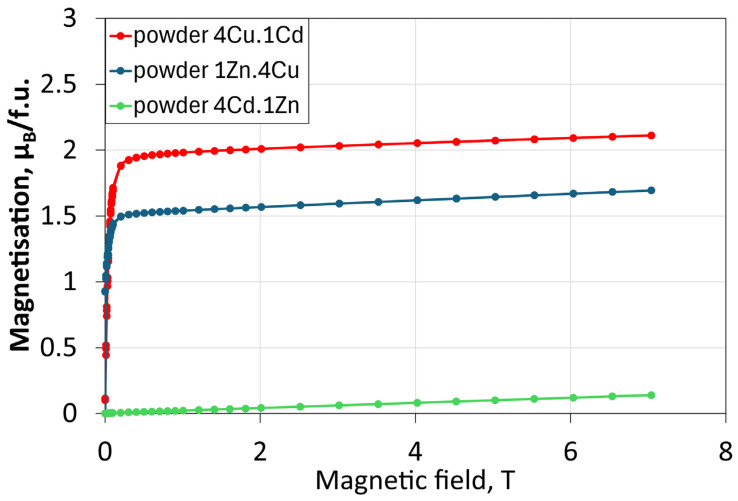
Magnetisation curves of a combination of powders at 300 K.

Figure 3 presents the dependence of magnetisation on the applied magnetic field for the powder mixtures used as fillers in the membranes: 4Cu.1Cd, 1Zn.4Cu, and 4Cd.1Zn. Measurements were carried out at 300 K. The 4Cu.1Cd powder exhibits the highest saturation magnetisation (2.3 μ_B_/f.u.), which is comparable to that of pure CuCr_2_Se_4_ [18]. In this mixture, both base spinel compounds display distinct magnetic behaviours. CuCr_2_Se_4_ is a ferromagnet with metallic conductivity. Its high Curie temperature (T_C_ ≈ 460 K, θ_CW_ ≈ 465 K) indicates very strong ferromagnetic coupling and suggests the presence of additional magnetic moment interactions localised on chromium ions, mediated by charge carriers [29,30]. In contrast, CdCr_2_Se_4_ is a ferromagnetic *p*-type semiconductor with T_C_ ≈ 130 K and θ_CW_ ≈ 200 K. At 300 K, the CdCr_2_Se_4_ exhibits paramagnetic properties [31]. Increasing temperature gradually disrupt spin homogeneity, and once the Curie temperature is reached, magnetic homogeneity is lost as the thermal energy exceeds the magnetic interaction energy.

The same phenomenon is observed for 1Zn.4Cu powder. In this mixture, the ferromagnetic component (CuCr_2_Se_4_) coexists with ZnCr_2_Se_4_, a spinel compound exhibiting different physicochemical and magnetic properties. ZnCr_2_Se_4_ is a *p*-type semiconductor with a helical antiferromagnetic (AFM) order below the Néel temperature (*T*_N_ = 20 K), accompanied by a strong ferromagnetic (FM) component, as evidenced by a large positive Curie-Weiss temperature (θ_CW_) of 115 K [32,33,34]. The magnetic saturation of the 1Zn.4Cu powder is approximately 1.8 μ_B_/f.u. For both the 4Cu.1Cd and 1Zn.4Cu mixtures, the magnetisation curves exhibit similar shapes, confirming the ferromagnetic character of the investigated samples. Magnetic saturation is achieved at very low magnetic fields. The higher magnetic saturation observed for 4Cu.1Cd compared to 1Zn.4Cu results from the fact that, in ferromagnets, exchange interactions do not vanish at the Curie temperature. Instead, they persist and continue to reinforce the magnetisation.

In contrast, the 4Cd.1Zn consists of two paramagnetic compounds: CdCr_2_Se_4_ and ZnCr_2_Se_4_, both of which are paramagnets at 300 K. Consequently, the dependence of magnetic saturation on magnetic field is linear (Figure 3).

These findings confirm that the presence of CuCr_2_Se_4_ strongly dominates the overall magnetic response of the composites, while the Cd- and Zn-based components mainly contribute to the paramagnetic behaviour.

**Figure 4:**
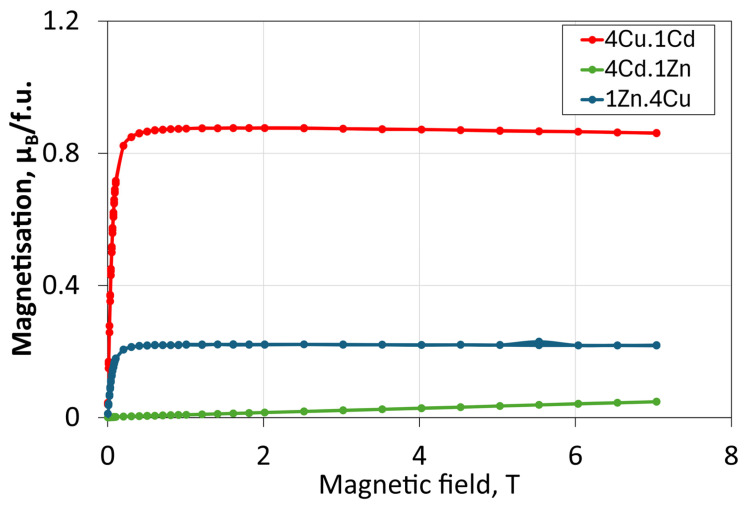
Magnetisation curves of membranes 4Cu.1Cd, 1Zn.4Cu, 4Cd.1Zn at 300 K.

Figure 4 presents the magnetisation isotherms for the three composite membranes filled with the respective mixture of spinel compounds: 4Cu.1Cd, 1Zn.4Cu, and 4Cd.1Zn. All measurements were performed at 300 K. The results reveal distinct magnetic responses that directly reflect the relative contributions of the individual ferromagnetic and paramagnetic components in each composite. The 4Cu.1Cd membrane, containing ferromagnetic CuCr_2_Se_4_ and paramagnetic CdCr_2_Se_4_ fillers, exhibits a saturation magnetisation of approximately 0.8 μB/f.u. The ferromagnetic response in the 4Cu.1Cd membrane is expected to enhance polarisation effects induced by the magnetic field within the polymer matrix, thereby promoting the alignment of ethanol molecules and polar water molecules during the dehydration process [25,35].

The 1Zn.4Cu membrane (Figure 4) also exhibits ferromagnetic properties, although with a lower saturation magnetisation of approximately 0.2 μB/f.u. The reduced magnetisation of the membranes compared to the corresponding powders may result from the partial “locking” of magnetic moments from CuCr_2_Se_4_ within the lattice of membranes. These magnetic moments do not fully achieve ferromagnetic ordering. Although ferromagnetism is preserved, the reduced magnetic response indicates weaker magnetic interactions, which may limit field-assisted transport during ethanol dehydration [18,36].

In contrast, the 4Cd.1Zn membrane (Figure 4) shows a linear magnetisation dependence, indicating its paramagnetic character, similar to that of the corresponding 4Cd.1Zn powder.

The observed magnetic behaviour is expected to significantly influence membrane performance in ethanol dehydration. Stronger magnetic ordering enhances local magnetic field gradients, thereby promoting the orientation of polar molecules within the membrane structure. This effect facilitates the sorption of water molecules and improves ethanol selectivity [25]. Therefore, the 4Cu.1Cd membrane, which exhibits the highest saturation magnetisation and the most potent ferromagnetic properties, is expected to demonstrate the best dehydration performance.

According to the theory presented by Pang et al. [37], exposing water to a magnetic field alters its macroscopic properties by modifying the distribution and microscopic structure of water molecules, including electron distribution, atomic displacements, and molecular polarisation. In a mixture containing a large proportion of a ferromagnetic phase, the presence of a domain structure is expected to play a significant role. Such a domain structure is characteristic of ferromagnets. As the magnetic field increases, domains oriented along the field direction expand. The boundaries between domains are not sharp. Instead, they consist of a transition region a few atomic layers thick. Under the influence of the magnetic field, these domains expand; however, in real samples, their walls eventually become pinned in metastable positions corresponding to local free-energy minima. Considering the structure of chromium-based spinels, the presence of vacancies, and the reported changes in the macroscopic properties of water, it is plausible that water and alcohol molecules—owing to their low mass—may become selectively trapped within such metastable positions.

The 1Zn.4Cu membrane, showing intermediate magnetic properties, should exhibit moderate activity, whereas the 4Cd.1Zn membrane, characterised by paramagnetism, is expected to perform the weakest. These findings highlight the crucial role of magnetic filler composition in determining both the magnetic and functional properties of hybrid membranes. They also indicate that optimising the CuCr_2_Se_4_-CdCr_2_Se_4_ ratio can effectively tune the magnetic response and dehydration efficiency of these membrane systems.

### 2.3. Pervaporation Performance

**Figure 5:**
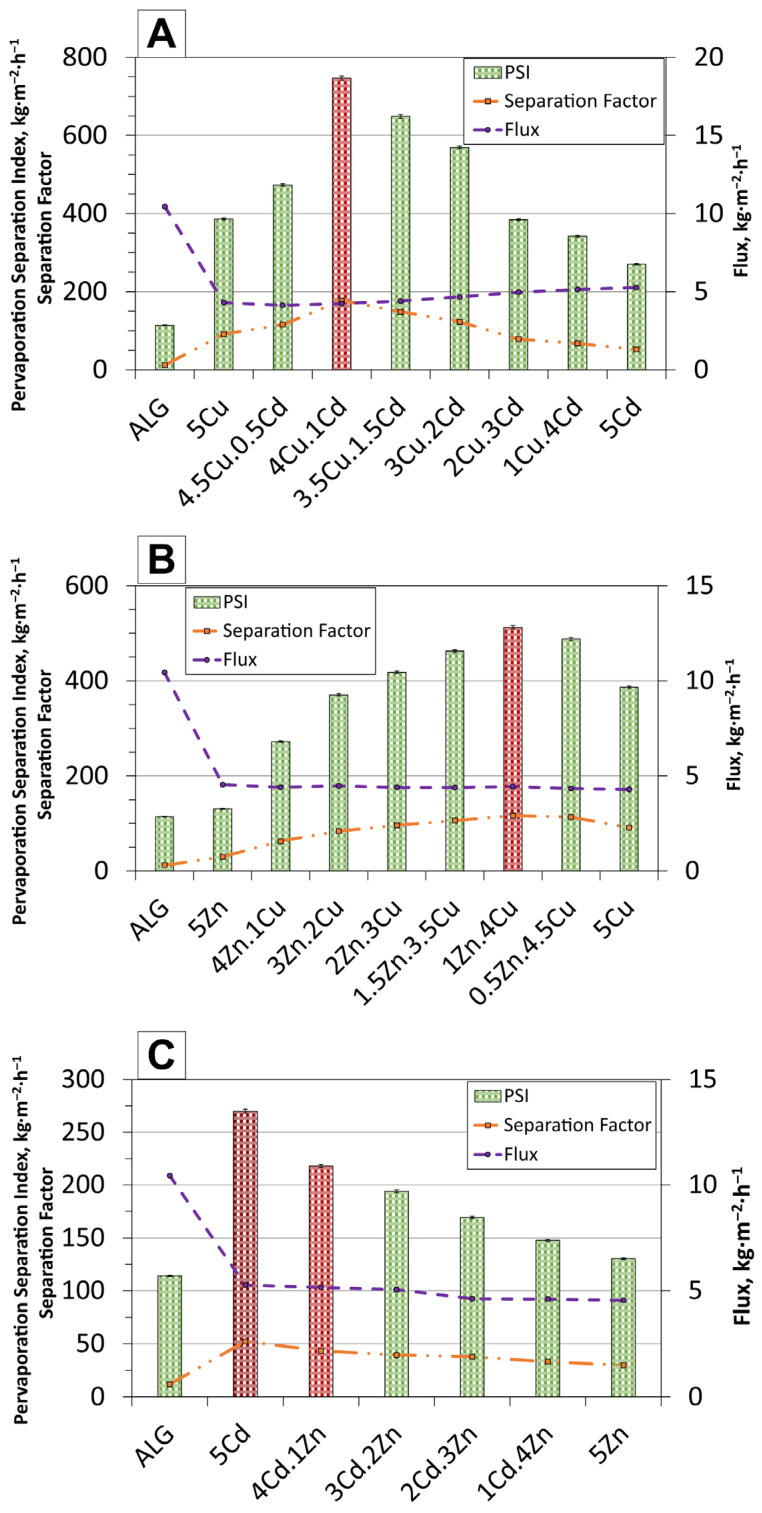
Variation in pervaporation flux, separation factor, and PSI for membranes containing different combinations of fillers: (**A**)—Cu.Cd, (**B**)—Zn.Cu, and (**C**)—Cd.Zn. Red graphs represent the best results for the series.

Figure 5A–C present the pervaporation results for alginate membranes containing various mixed powder compositions, along with comparisons to membranes filled with individual powders. For the Cu.Cd series, the membrane with the 4Cu.1Cd composition exhibits the highest PSI value (747 kg·m^−2^·h^−1^), demonstrating the best overall performance. Both the PSI and separation factor values increase with the Cu/Cd ratio up to the 4Cu.1Cd composition and then decrease at lower Cu contents. All membranes containing two different fillers outperformed those containing a single filler. In the CuCr_2_Se_4_-based series, membranes with 0.5 wt.% deviations in powder content were also tested to verify the observed trends near the optimal membrane compositions. In the Zn.In the Cu series (Figure 5B), a similar trend was observed: the membrane containing 4 wt.% CuCr_2_Se_4_ powder (1Zn.4Cu) showed the highest pervaporation performance, reaching the maximum PSI (513 kg·m^−2^·h^−1^) and separation factor (116). As in the Cu.Cd series, all membranes containing a combination of two powders performed better than those with only one filler. Conversely, in the Cd.Zn series, membranes with mixed fillers exhibited lower performance than those filled only with CdCr_2_Se_4_, but higher than those filled exclusively with ZnCr_2_Se_4_. The PSI and separation factor values gradually decreased with increasing ZnCr_2_Se_4_ content, making the 5Cd membrane the best-performing membrane in this series.

An intriguing relationship was observed between the flux and the separation factor. According to the commonly reported trade-off behaviour [38], an increase in the separation factor is usually accompanied by a decrease in total flux, and vice versa. This trend was clearly observed for the Cd.Zn and Zn.Cu series. However, in the Zn.Cu membranes showed a less pronounced effect, with nearly constant flux values across all samples. The Cd.Zn membranes exhibited the most distinct trade-off relationship. In contrast, the Cu.Cd membranes showed the opposite trend: the flux increased for all membranes containing two fillers. As shown in Table 2, the detailed flux data indicate an increase when the filler ratio was changed from 4.5Cu.0.5Cd to 4Cu.1Cd. Interestingly, for these first two compositions (4.5Cu.0.5Cd and 4Cu.1Cd), the separation factor also increased, reaching a maximum value of 177.24 for the 4Cu.1Cd membrane.

**Figure 6:**
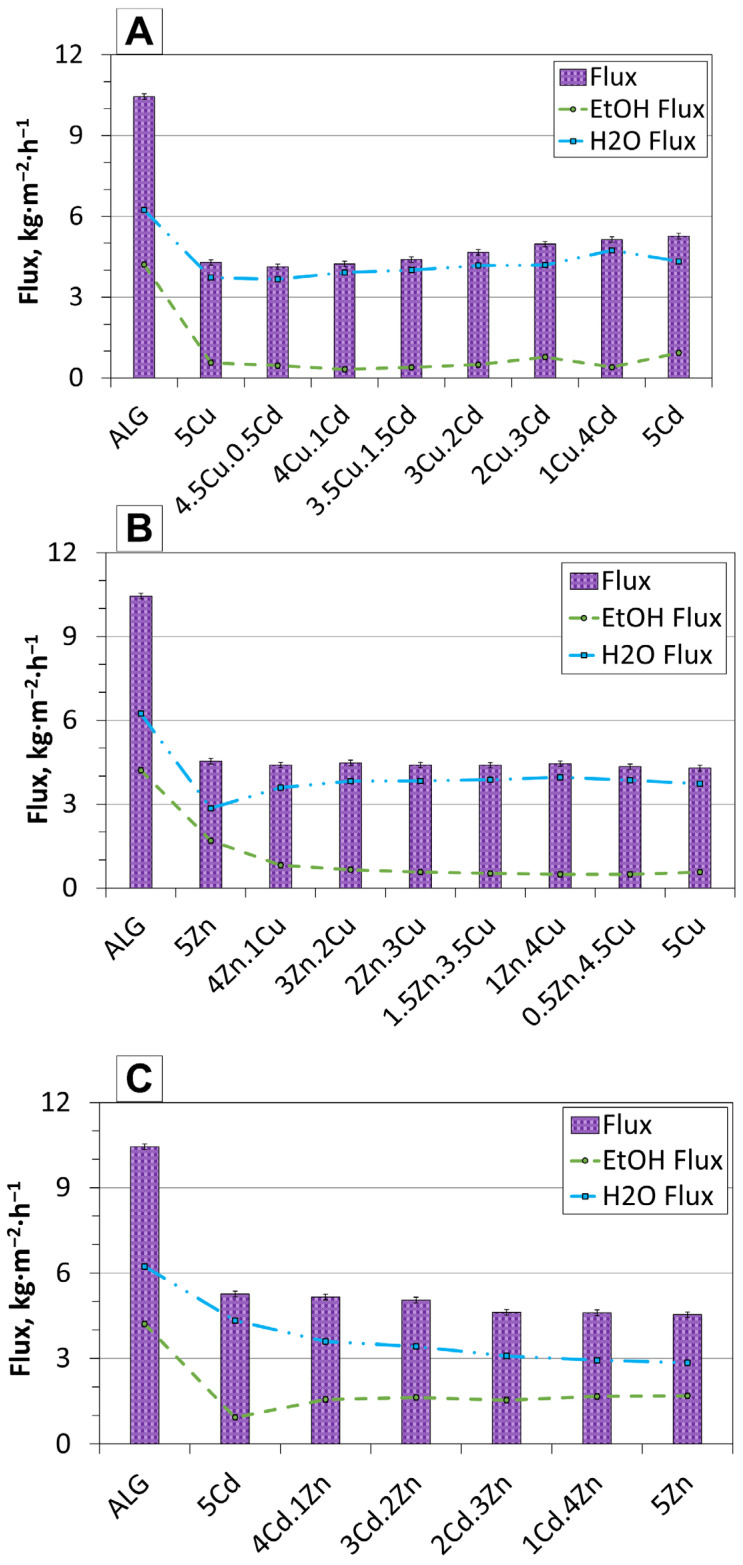
Pervaporation fluxes of total permeate, ethanol (EtOH), and water (H_2_O) for alginate-based membranes containing different combinations of fillers: (**A**)—Cu.Cd, (**B**)—Zn.Cu, and (**C**)—Cd.Zn.

To explain this unexpected behaviour in which both flux and selectivity improved simultaneously, an analysis of the total flux and the individual water and ethanol fluxes was conducted (Figure 6A–C). The typical relationship between component fluxes and the total flux is illustrated in Figure 6B for Zn.Cu membranes. While the total flux remains nearly constant, the individual water and ethanol fluxes change inversely: as the water flux increases, the ethanol flux decreases. In this case, magnetic interactions among the fillers, the membrane matrix, and the feed components affect only the ratio of the two fluxes. The membrane exhibits a stronger affinity for water molecules, leading to partial blocking of ethanol transport; however, the total flux remains unchanged because the interactions are not strong enough to significantly enhance overall permeation.

In contrast, the results for the Cu.Cd membranes show that the best-performing samples exhibit an increase in the overall flux, even though the ethanol flux decreases. This behaviour can be attributed to strong magnetic interactions between the fillers and water molecules. As suggested by Pang et al. [37], a magnetic field can alter the structure and arrangement of water molecules, thereby affecting their transport behaviour. In materials containing magnetic domains, water molecules may interact more strongly with these regions, enabling them to pass through the membrane more readily. The opposite trend is observed for the Cd.Zn membranes (Figure 6C), where the ethanol flux increases while the water flux decreases; nevertheless, the total flux continues to rise. It suggests that ethanol molecules are not effectively repelled, and water molecules are only weakly attracted, allowing ethanol to pass through the membrane more readily.

The higher PSI and separation coefficient values obtained for the 4Cu.1Cd membrane can be interpreted in terms of its structural and magnetic characteristics discussed previously. The uniform dispersion of chromite particles and the absence of agglomerates ensure a continuous polymer phase with well-defined water diffusion channels [39]. This morphology facilitates efficient sorption and transport of water molecules through evenly distributed magnetic domains, while limiting the formation of non-selective defects and aggregates that could promote ethanol permeation. Such structural uniformity directly contributes to the observed increase in both total flux and selectivity. Moreover, the strong ferromagnetic response of the 4Cu.1Cd system may enhance local magnetisation within the polymer matrix, thereby improving the orientation of polar water molecules and stabilising hydrogen-bond structures at the filler-polymer interface [25,40]. These magnetically induced interactions enhance the membrane’s affinity for water, leading to increased water flux and improved ethanol rejection. The bonding between ferromagnetic domains and the polar polymer network thus generates additional driving forces for selective transport, explaining why the performance of the 4Cu.1Cd membrane exceeds the efficiency expected based solely on morphological considerations.

On the other hand, membranes containing zinc chromite, such as 1Zn.4Cu and 4Cd.1Zn, exhibit less favourable structural and magnetic configurations. The presence of aggregated filler clusters and weaker magnetic ordering reduces the effectiveness of these compounds. In the 1Zn.4Cu membrane, partial clustering and reduced magnetisation limit the formation of uniform, selective channels, resulting in moderate fluxes and only modest increases in selectivity. The 4Cd.1Zn membrane, characterised by a non-uniform morphology and a largely paramagnetic reactivity, exhibits even weaker interactions between water and filler and less selective transport channels, resulting in the lowest pervaporation efficiency among the studied combinations.

### 2.4. Comparison of Pervaporation Performance with the Literature

Most studies on the pervaporation of ethanol–water mixtures indicate a clear trade-off between permeation flux and separation factor, where an increase in permeability comes at the cost of selectivity [14,38]. In a study published by Zhan et al. [13], the authors observed that the incorporation of hydrophobic graphene into a poly(vinyl alcohol) membrane increased the separation factor to approximately 1400 but significantly reduced the flux, while the use of graphene oxide fillers led to a higher flux at the cost of selectivity. A similar phenomenon was demonstrated by Asmaa et al. [41]. The authors also filled a poly(vinyl alcohol)-based membrane with silver nanoparticles, which increased the water permeation flux but decreased the separation factor. Such trade-off relationships between flux and selectivity are widely reported in the literature [12,14,25,38,42]. As a result, scientists began exploring hybrid and composite membranes to overcome this trade-off. In fact, some specially engineered materials have shown simultaneous improvements in both flux and selectivity. For example, polydimethylsiloxane mixed-matrix membranes filled with ZIF-L nanosheets, as reported by Pei et al. [43], showed simultaneous increases in ethanol and water flux and in the separation factor. In another study, Liang et al. [44] researched an innovative asymmetric PDMS/PVDF composite membrane that showed an increase in separation factor with increasing flux, effectively overcoming the typical trade-off between permeability and selectivity. Moreover, Yi et al. [45] used advanced nanostructured membranes. In their study, the authors demonstrated that ultra-thin membranes with a covalent organic framework (COF) created by integrating COF-TbTG and sulfobutylether-β-cyclodextrin (SCD) nanolayers into laminar structures developed more water-selective channels as more SCD was added. This led to a continuous rise in flux. Additionally, incorporating SCD improved the membrane separation factor, effectively overcoming the typical trade-off. In this context, the Cu.Cd membrane is noteworthy because, although its absolute flux and separation factor are not the highest among those reported, it uniquely achieves simultaneous improvements in both. This behaviour breaks away from the typical flux-selectivity trade-off observed in most membranes and aligns with a growing research trend aimed at overcoming this limitation. Such performance indicates that the Cu.Cd membrane design successfully balances permeability and selectivity, resulting in a rare combination of enhanced flux and improved separation efficiency.

## 3. Materials and Methods

### 3.1. Materials

Sodium alginate (Brookfield viscosity 350–550 mPas, c = 1 wt.% at 20 °C) and ethanol (95 wt.%, extra pure) were purchased from ACROS ORGANICS. Calcium chloride (purity ≥ 96%) was purchased from Fisher Scientific, Waltham, MA, USA. All chemicals were used as received without any further purification. The ZnCr_2_Se_4_, CdCr_2_Se_4_ and CuCr_2_Se_4_ powders were synthesised using pure elements (Cu, Cd, Zn, Cr and Se, Sigma Aldrich, St. Louis, MO, USA, 5 N purity) by the high-temperature sintering of powders.

### 3.2. Preparation of Selenide Chromites

Selenium chromites were prepared under controlled conditions to obtain well-crystallised phases with magnetism suitable for the preparation of composite membranes. Materials with an appropriate composition of ACr_2_Se_4_ (A = Cd, Cu, Zn) were obtained by sintering powder at high temperature using a two-step method. Firstly, binary selenides CdSe, CuSe, ZnSe and Cr_2_Se_3_ were synthesised by sintering stoichiometric mixtures of elemental precursors Cd, Cu, Zn, Cr and Se at 1073 K for 10 days. In the next step, stoichiometric combinations of these binary compounds were mixed and subjected to two sintering cycles at 1173 K, each lasting 240 h, to obtain ACr_2_Se_4_ phases according to the reaction scheme:

ASe + Cr_2_Se_3_ ⟶ ACr_2_Se_4_, where A = Cd, Cu, Zn

Both syntheses were carried out in silica ampules (outer diameter, 20 mm; length, ~200 mm). The tubes were evacuated to ~10^−5^ mbar using a turbomolecular pumping system and flame sealed using an O_2_/H_2_ burner. After sintering, the products were cooled and then finely ground in an agate mortar.

### 3.3. Membrane Preparation

The hybrid alginate membranes containing mixtures of zinc and cadmium, zinc and copper or cadmium and copper chromite selenide powders were prepared by dissolving 4.57 g of sodium alginate in 300 mL of deionised water. The polymer solution was continuously stirred using a magnetic stirrer (IKA, Staufen, Germany) for 24 h to ensure complete dissolution. Following this, the solution was filled with 5 wt.% of inorganic fillers, comprising specific weight ratios of appropriate chromite selenide powders, as detailed in Table 3. An ultrasonic bath then homogenised the mixture for 1 to 3 h. This step was performed to promote uniform dispersion of the fillers throughout the alginate matrix. The resulting mixture was carefully cast onto wax-coated Petri dishes and dried at 22 °C for 48 h under ambient conditions.

After drying, the membranes were cross-linked by immersing them in 62.5 mL of a 2.5 wt.% CaCl_2_ solution for 2 h to improve their structural integrity and water resistance. After cross-linking, the membranes were removed from the Petri dishes and thoroughly washed with deionised water to remove residual calcium ions. The thickness of the obtained membranes was measured at 50 points using a thickness gauge, and the average value for all samples was approximately 20–30 µm.

### 3.4. Physicochemical Characterisation

To correlate separation behaviour with material properties, additional structural and chemical analyses were performed. Morphological features of both pristine and magnetic membranes were investigated by HR-SEM (Zeiss Supra 35, Carl Zeiss Microscopy GmbH, Jena, Germany) at magnifications up to 50,000×. Surface details were analysed using SE and In-Lens detectors, with gold sputtering applied to enhance conductivity. The elemental composition of the chromite selenide powder phases was verified by EDS (Thermo Scientific UltraDryThermo Fisher Scientific, USA), with data processed in Pathfinder software. Quantitative image analysis was performed using Python-based (version 3.12.7) workflows, USA (scikit-image (version 0.24.0), NumPy (version 1.26.4), pandas (version 2.2.3), SciPy (version 1.12.0), Matplotlib (version 3.8.4), and Seaborn (version 0.13.2)) to determine particle size distributions, packing density, and spatial heterogeneity. Outputs were summarised as histograms, probability distributions, and density maps. Furthermore, magnetic characterisation was conducted on individual powders and magnetic membranes using a SQUID magnetometer (Quantum Design XL-7,Quantum Design, Inc., San Diego, CA, USA) under applied fields up to 7 T over the temperature range 10–300 K. Additionally, membrane thickness measurements were performed at 50 points using a thickness gauge (Elmetron MG-401Elmetron Sp. z o.o., Zabrze, Poland). 

### 3.5. Pervaporation Process

Membrane separation experiments were conducted using a specially designed pervaporation system. The feed solution consisted of 1 dm^3^ ethanol–water mixtures with an ethanol concentration of 95 wt.%, as well as pure water for reference (the ethanol concentrations of feed, permeate, and retentate were determined by gas chromatography). The liquid was continuously circulated through the separation chamber, where the membrane had an effective surface area of 4.1 × 10^−4^ m^2^. To maintain a constant feed composition, the retentate stream was recirculated back to the feed tank. The permeate vapours were collected in traps cooled with liquid nitrogen to avoid reevaporation, while the system pressure was controlled by a vacuum pump and monitored with a vacuum gauge. Throughout all experiments, the vacuum level was maintained at 2.9–3.5 mbar. A total of five pervaporation samples were performed, and the average separation parameters were calculated from them. Both the feed temperature and the overall process were conducted at room temperature. After the experiment, the condensed permeate was thawed and weighed using an analytical balance to determine the total flux. A comprehensive description of the experimental setup was provided in our previous studies [18].

Performance evaluation was based on the pervaporation separation index (PSI), which simultaneously combines flux and selectivity [38,46]:

(1) $$PSI=J\left(\alpha_{AB}-1\right)\left[\frac{kg}{m^{2}\cdot h}\right]$$

where J is the total permeate flux $\left[\frac{kg}{m^{2}\cdot h}\right]$, α_AB_ is the separation factor, determined from the relative mass fractions of components in the feed (x) and permeate (y) [47,48]:

(2) $$\alpha_{A/B}=\;\frac{y_{A}/y_{B}}{x_{A}/x_{B}}$$

The partial flux of component *i* (water, ethanol, total permeate) was calculated as [46,47]:

(3) $$J_{i}=\frac{m_{i}}{A\cdot t}\left[\frac{kg}{m^{2}\cdot h}\right]$$

where m_i_ is the weight of the component in the permeate [kg], A is an effective membrane area 4.10·10^−4^ [m^2^], t is the permeation time [h].

## 4. Conclusions

This study demonstrates that hybrid alginate membranes incorporating two-component chromium seledine fillers offer a versatile approach to tuning membrane morphology, magnetic reactivity, and mass transport properties during ethanol dehydration by pervaporation. The combination of copper and cadmium chromites (CuCr_2_Se_4_ and CdCr_2_Se_4_) in a 4:1 ratio (4Cu.1Cd) provided the most favourable performance among all tested compositions, achieving a pervaporation separation index (PSI) of 747 kg·m^−2^·h^−1^. The exceptional behaviour of the 4Cu.1Cd membrane, characterised by a simultaneous increase in total flux and selectivity, can be attributed to synergistic structural and magnetic effects. The uniform dispersion of the filler, coupled with strong ferromagnetic ordering, promotes the formation of continuous water-selective transport channels and enhances interfacial polarisation at the membrane-feed interface. These magnetically induced interactions strengthen the membrane’s affinity for water molecules, improving selective sorption and diffusion while limiting ethanol permeation.

In contrast, membranes containing CuCr_2_Se_4_ and ZnCr_2_Se_4_ were characterised by less favourable morphology, including particle aggregation and filler-free regions, weaker magnetic reactivity, and a moderately reduced separation efficiency. Moreover, membranes filled with ZnCr_2_Se_4_ and CdCr_2_Se4 did not provide the expected magnetic response. Both compounds are paramagnetic at room temperature. Magnetometric analyses also revealed that the polymer matrix can, to some extent, scale down or even suppress ferromagnetic ordering, emphasising the importance of selecting appropriate filler pairs and their proportions to achieve synergistic behaviour.

Overall, hybrid alginate membranes incorporating two-component chromium selenides represent a sustainable and environmentally friendly strategy for enhancing ethanol dehydration efficiency. The superior performance of the 4Cu.1Cd membrane demonstrates that tailoring magnetically responsive membrane structures and interfacial polarisation can effectively mitigate the conventional flux-selectivity trade-off while maintaining mechanical integrity and functional stability.

## Figures and Tables

**Table 1:** Statistical parameters of particle diameter and nearest-neighbour distance distributions.

Membrane	Parameter	Median (µm)	Mean (µm)	Standard Deviation (µm)	Interquartile Range (µm)
4Cu.1Cd	Particle Size	3.39	4.05	2.72	2.09–5.18
4Cu.1Cd	Neighbour Distance	9.62	10.95	5.91	6.40–14.34
1Zn.4Cu	Particle Size	3.01	3.82	2.84	1.95–4.74
1Zn.4Cu	Neighbour Distance	10.21	11.53	6.13	6.82–15.07
4Cd.1Zn	Particle Size	2.99	3.79	2.74	1.95–4.79
4Cd.1Zn	Neighbour Distance	10.97	12.32	7.01	6.66–16.52

**Table 2:** Detailed values of flux for Cu.Cd membranes.

Type of Membrane	5Cu	4.5Cu.0.5Cd	4Cu.1Cd	3.5Cu.1.5Cd	3Cu.2Cd	2Cu.3Cd	1Cu.4Cd	5Cd
Flux value [kg·m^−2^·h^−1^]	4.29	4.12	4.24	4.40	4.67	4.97	5.14	5.27

**Table 3:** Table presenting the weight ratios of zinc, cadmium, and copper chromite selenide powders used as magnetic membrane fillers.

Type of Membrane	ZnCr_2_Se_4_	CdCr_2_Se_4_	CuCr_2_Se_4_
ALG	x	x	x
5Cu	x	x	5 wt.%
4.5Cu.0.5Cd	x	0.5 wt.%	4.5 wt.%
4Cu.1Cd	x	1 wt.%	4 wt.%
3.5Cu.1.5Cd	x	1.5 wt.%	3.5 wt.%
3Cu.2Cd	x	2 wt.%	3 wt.%
2Cu.3Cd	x	3 wt.%	2 wt.%
1Cu.4Cd	x	4 wt.%	1 wt.%
5Cd	x	5 wt.%	x
4Cd.1Zn	1 wt.%	4 wt.%	x
3Cd.2Zn	2 wt.%	3 wt.%	x
2Cd.3Zn	3 wt.%	2 wt.%	x
1Cd.4Zn	4 wt.%	1 wt.%	x
5Zn	5 wt.%	x	x
4Zn.1Cu	4 wt.%	x	1 wt.%
3Zn.2Cu	3 wt.%	x	2 wt.%
2Zn.3Cu	2 wt.%	x	3 wt.%
1.5Zn.3.5Cu	1.5 wt.%	x	3.5 wt.%
1Zn.4Cu	1 wt.%	x	4 wt.%
0.5Zn.4.5Cu	0.5 wt.%	x	4.5 wt.%

## Data Availability

The original contributions presented in this study are included in the article. Further inquiries can be directed to the corresponding author.
